# Extracorporeal membrane oxygenation for pulmonary haemorrhage

**DOI:** 10.1136/bcr-2025-265755

**Published:** 2025-12-18

**Authors:** Yuan Feng, Yinfei Zhou, Xi Peng, Rong Huang

**Affiliations:** 1 Department of Pediatrics, Xiangtan Central Hospital, Xiangtan, Hunan, China

**Keywords:** Mechanical ventilation, Trauma, Neonatal and paediatric intensive care, Pediatrics

## Abstract

Extracorporeal membrane oxygenation (ECMO) technology has relative contraindications in haemorrhagic disease but remains an effective therapeutic option for the treatment of pulmonary haemorrhage associated with respiratory failure. We report a critically ill case of a child with pulmonary haemorrhage and respiratory failure due to unintentional injury that was successfully treated with ECMO running at a low dose of heparin. An anticoagulation strategy with appropriate doses of heparin may improve the success of ECMO techniques in the treatment of severe pulmonary haemorrhage.

## Background

 Extracorporeal membrane pulmonary oxygenation (ECMO) is a new technology that provides cardiac and respiratory support outside the human body and has become an effective treatment for severe cardiopulmonary failure. Currently, ECMO technology is used in paediatrics primarily for acute respiratory distress syndrome (ARDS), respiratory failure and heart failure due to infectious lung disease, with rare cases of pulmonary haemorrhage in both children and adults and relatively few cases of ECMO-treated pulmonary haemorrhage.^1^

Pulmonary haemorrhage is a low incidence acute respiratory disease that accounts for about 5% of all coughing-up-blood cases and is even rarer in children but has a mortality rate of up to 38%.^2^ The case we report is a serious unintentional injury. In the presence of active bleeding from the lungs and fracture site, routine anticoagulation during ECMO therapy carries the risk of exacerbating the bleeding and endangering life. Special attention must be paid to the dosage and timing of anticoagulant therapy drugs. Accordingly, a synopsis of the treatment of this case is hereby presented, along with the sharing of our experience. It is our hope that this will assist clinicians in the utilisation of ECMO for the treatment of pulmonary haemorrhage, in conjunction with reasonable anticoagulation therapy, thereby enhancing the efficacy of treatment and improving the overall cure rate.

## Case presentation

An early adolescent girl fell down a 3-m-high hill near her home, landing on her left foot and simultaneously striking her forehead. The impact resulted in exposed bone and bleeding at her left ankle, accompanied by severe pain and limited mobility. She was immediately admitted to a local hospital for debridement and wound closure of the injured ankle. The day before admission, she presented with chest pain, chest tightness, shortness of breath, cyanosis, palpitations and dizziness. 4 hours earlier, she had developed multiple haemoptysis and was taken by ambulance from a local hospital to our trauma centre for a CT scan of her lungs and then admitted to the paediatric intensive care unit for treatment.

Admission physical examination: temperature 37.8℃, pulse rate 140 beats per minute, respiratory rate 40 breaths per minute, weight 84 kg, blood pressure 74/49 mm Hg, mental clarity, obese body type, periorbital bruises, double eyelid oedema, lips cyanosis, respiratory urgency, visible nasal flap and triple concave sign, both lungs’ respiratory sounds thick, and wet rhonchi can be heard. The left lower limb was immobilised in a plaster cast; the exposed part of the ankle was swollen, and the skin was bruised; the dorsalis pedis arteries of both lower limbs had normal pulsations, and the activities of the upper limbs and the right lower limb were normal.

Auxiliary examination in the local hospital: X-ray of the left lower limb in the outside hospital showed fracture of the left middle femur, open comminuted fracture of the left talus and collapse of the ankle joint surface; head CT showed comminuted fracture of the right nasal bone and bilateral maxillary and pterygoid sinus effusion; lung CT showed no apparent abnormality.

**Figure 1:**
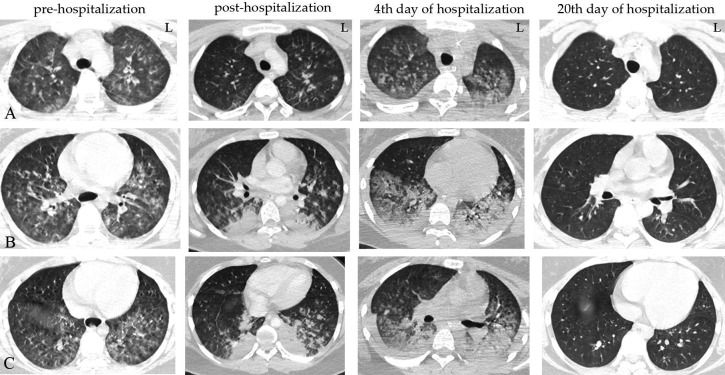
. The child's prehospital lung CT showed diffuse lesions in both lungs, and the possibility of hemorrhage from lung injury was considered high; posthospital follow-up lung CT showed pulmonary hemorrhage consistent with bilateral pulmonary hemorrhage (the lower lobes of both lungs were more solid than before); on day 4, lung CT showed exudative lesions in both lungs (which were more numerous than before) and a small amount of pleural effusion on the right side; and on day 20, lung CT showed no apparent abnormality. (Note: row A is the upper lobe of the lung, row B is the middle lobe of the lung, and row C is the lower lobe of the lung).

Our emergency lung CT showed diffuse lesion shadows in both lungs, considering the possibility of haemorrhage in lung injury, not excluding co-infection, and suggesting review after treatment (figure 1).

## Investigations

### Blood biochemical parameters

Dynamic monitoring of liver and kidney function, cardiac enzymes, electrolytes, troponin T, B-type natriuretic peptide and procalcitonin levels during the hospitalisation of the children was basically in the normal range. Changes in routine blood monitoring, blood gas analysis, coagulation function, D-dimer, C reactive protein and blood lactate results are shown in table 1.

**Table 1:** Results of routine laboratory indicator monitoring

Testing indicators	Routine blood count			Blood gas analysis			Coagulation routine			C reactive protein (mg/mL)
	White blood cells (×10^9^/L)	Haemoglobin (g/ L)	Platelets (×10^9^/L)	Alveolar-arterial oxygen gradient (mm Hg)	Arterial oxygen pressure/arterial carbon dioxide pressure (mm Hg)	Blood lactate (mmol/L)	Activated partial thromboplastin time (s)	Fibrinogen (g/L)	D-dimer (ug/mL)	
Hospitalisation	23.25	84.0	200.0	56	35/47	4.9	3.82	3.27	3.73	96.66
Before ECMO treatment	17.46	82.0	138.0	620	42/41	1.1	40.5	3.47	4.53	–
After ECMO treatment	17.29	104.0	107.0	186	57/34	1.7	40.2	3.23	5.23	69.26
Days 3–4	14.19	90.0	90.0	157	74/43	2.2	32.5	2.07	5.85	10.89
Before ECMO withdrawal	18.49	93.0	100.0	155	79/31	0.8	60.5	2.34	4.17	–
After ECMO withdrawal	22.59	102.0	108.0	229	76/47	0.6	36.8	3.52	3.64	79.45
Preoperative	14.3	98.0	195.0	142	82/35	0.5	41.7	2.56	4.28	43.98
Postoperative	11.75	96.0	271.0	120	110/43	0.7	28.7	4.71	4.01	44.03
Ventilator withdrawal	9.0	101.0	406.0	82	99/41	0.5	30.1	4.64	4.94	13.7

ECMO, extracorporeal membrane pulmonary oxygenation.

### Imaging

**Figure 2:**
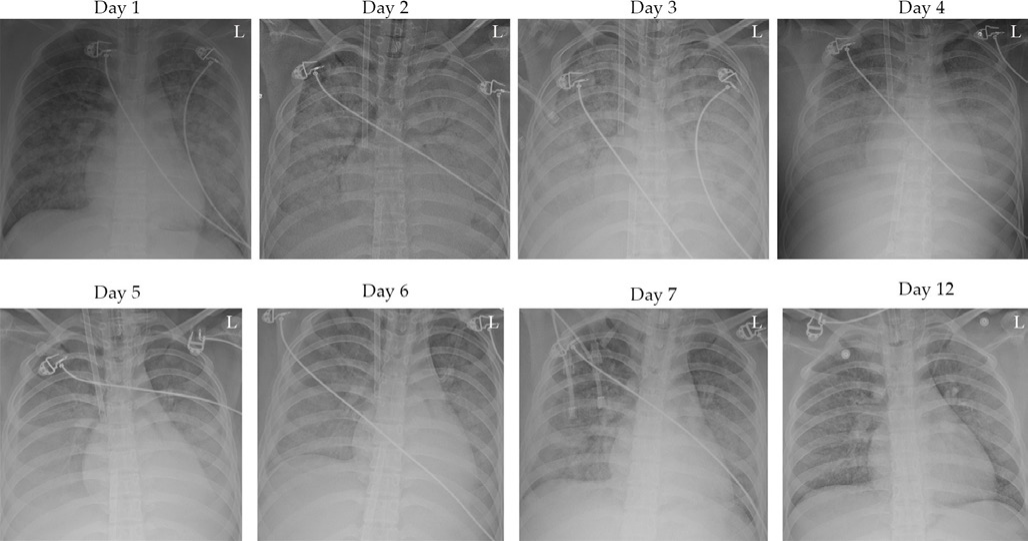
. Chest X-rays of the cases at different time points showed gradual absorption of the exudative lesions in the lungs after day 4-5, and the chest X-rays of the cases at day 12 were essentially normal.

**Figure 3:**
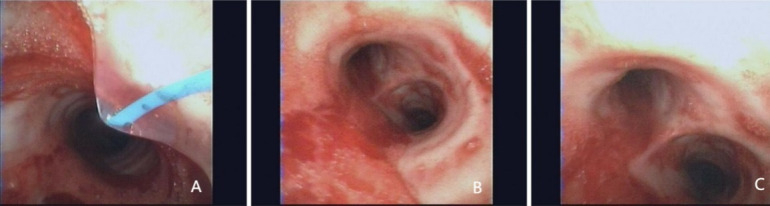
. Bedside bronchoscopy was performed at three distinct time points for this particular case. (A and B: the initial bronchofiberscopy and subsequent bronchofiberscopy revealed increased blood secretion in the endotracheal tubes, bilateral congestion of the mucosa of grade 1-4 bronchi, and a substantial amount of blood secretion in the airways, with a greater prevalence on the left side. C: the third fiberoptic bronchoscopy revealed a decrease in the presence of bloody fluid in the airway.)

The case underwent four lung CTs (figure 1), multiple bedside lung radiographs (figure 2) and three bedside bronchoscopies (figure 3) on admission and during hospitalisation.

## Differential diagnosis

We used a combination of enhanced CT of the lungs (figure 1) and bedside bronchoscopy (figure 3) to identify pulmonary vascular malformations and pulmonary emboli.

## Treatment

The case received mechanical ventilation immediately on admission, along with comprehensive symptomatic supportive therapy such as volume expansion for anti-shock, raising blood pressure and maintaining a stable internal environment. Various haemostatic agents were used to stop bleeding, and respiratory parameters were adjusted to control pulmonary haemorrhage.

Bedside bronchoscopy was completed at the 12th hour of admission (figure 3)**,** confirming the diagnosis of pulmonary haemorrhage, and alveolar lavage was performed. The combination of transfusion of large amounts of red blood cells, plasma, pressor and haemostatic agents was ineffective, and the child’s persistent intratracheal haemorrhage and decreasing blood oxygenation led to an urgent multidisciplinary consultation (MDT) at the hospital, which recommended ECMO treatment.

ECMO extracorporeal circulation treatment was performed at 14 hours of admission, and the V-V ECMO mode was adopted, and the adjustment of ECMO and ventilator parameters during the rescue period is shown in table 2.

**Table 2:** ECMO parameters and ventilator parameter settings

	ECMO					Ventilator				
Time	Model	Pump blood flow (L/min)	Rotational speed (r/min)	Gas flow/FiO_2_ (L/min)	TEG (CI)	Mode	VT (mL)	Respiratory rate (breaths per minute)	FiO_2_	Positive end-expiratory pressure (cmH_2_O)
Hospitalisation	–	–	–	–	–	PC	380	20	0.6	10
3 hour before ECMO	–	–	–	–	–	PC	380	20	1	12
3 hour after ECMO	V-V	4.41	3515	5.0 (1.0)	2.7	PC	200	12	0.4	10
Day 4	V-V	4.7	3545	5.5 (1.0)	1	PC	150	12	0.6	11
24 hours before ECMO withdrawal	V-V	3.45	2755	5.0 (1.0)	−4.2	PC	250	12	0.4	11
ECMO withdrawal	V-V	3	2470	3.0 (1.0)	−2.4	PC	360	12	0.4	8
Preoperative	–	–	–	–	–	PC	390	20	0.45	8
Postoperative	–	–	–	–	–	PC	410	20	0.4	8
Ventilator withdrawal	–	–	–	–	–	SIMV	400	15	0.35	5

FiO_2_ concentrations are expressed as decimals (1.0 is 100%); TEG monitoring: CI is the coagulation composite index (normal range −3.0 to 3.0); Ventilator PC mode VT values are calculated values based on other parameters.

ECMO, extracorporeal membrane oxygenation; FiO_2_, fractional inspired oxygen; PC, pressure control; TEG, thromboelastography; VT, tidal volume.

## Outcome and follow-up

In the first 4 days after admission, bedside chest radiography (figure 2) and bedside bronchoscopy showed significant pulmonary haemorrhage, and based on monitored thromboelastography (TEG), which ensured that the TEG coagulation composite index was between +3 and −3 (table 2). We administered subcutaneous injections of 3200 IU of low-molecular-weight heparin sodium every 12 hours for anticoagulant therapy. Simultaneously, the total fluid pumping rate was controlled at 120 mL/hour to 200 mL/hour according to the calculation of the daily in and out volume, and blood products such as plasma, platelets or cold precipitate were transfused to try to maintain the platelet count above 100×10^9^/L and the fibrinogen between 2.0 and 3.5 g/L.

On the fifth day, a significant reduction of bloody fluid in the tracheal tube of the case was observed, and the TEG showed a hypocoagulable state. We had another MDT consultation and recommended the addition of anticoagulant therapy with low-dose intravenous pumped heparin sodium (1.25–2.5 IU/kg/hour) to subcutaneous low-molecular-weight heparin sodium.

On the sixth day, no significant bleeding was seen on tracheal intubation, and bedside chest radiography showed better absorption of exudative lesions in both lungs than before. The third bedside bronchoscopy showed a significant decrease in bilateral lung and airway bleeding compared with the previous period. ECMO treatment was discontinued on the seventh day.

On the ninth day, the case underwent incision and reduction plate and screw internal fixation of the left mid-femur fracture, internal fixation of the left talus screw, and two-stage suturing of the left ankle joint trauma under general anaesthesia.

On the 12th day, the bedside lung X-ray showed that the pulmonary exudate was basically absorbed compared with the previous one, and the ventilator parameters were stable, so the ventilator was withdrawn.

On the 20th day, the lung CT was repeated and showed almost normal.

On the 24th day, the case was discharged from the hospital in an improved condition.

## Discussion

This case report describes a child who was obese and experienced blunt chest trauma due to a fall from a height, despite the absence of overt trauma at the time. Therefore, the initial step is to discuss the aetiology or pathophysiology of the pulmonary haemorrhage in this case. Mechanisms of traumatic pulmonary haemorrhage were explored in a multicentre prospective study^3^ . When various strong external forces act on the chest wall, the volume of the thoracic cavity decreases, causing pressure damage to the lung tissue. When the external forces are removed, the resulting negative intrathoracic pressure exacerbates the original tissue damage, leading to rupture of alveolar capillaries and bleeding, causing pulmonary haemorrhage. Given the greater elasticity of the chest bones in children compared with adults, it is hypothesised that the underlying cause or mechanism of pulmonary haemorrhage in this case was due to injury to the small blood vessels in the lungs, resulting from severe high-pressure shock caused by their own greater body weight. However, further research is necessary to substantiate this finding and to expand the range of case studies and models under review.

Second, in light of the clinical features of this case, the primary focus of our diagnostic efforts is to exclude two distinct pathologies: pulmonary vascular malformations and pulmonary embolisms. Pulmonary haemorrhage due to pulmonary vascular malformation is predominantly attributable to the rupture of pulmonary blood vessels, precipitated by external factors. In contrast, pulmonary embolism may result from various aetiologies, including coagulation abnormalities due to trauma. Consequently, in clinical practice, the performance of a pulmonary angiography CT scan is instrumental in identifying the presence of a pulmonary vascular malformation and a pulmonary embolism. In this case, due to unique circumstances, we were unable to perform pulmonary angiography CT in a timely manner. The condition posed a heightened risk due to the substantial amount of bleeding resulting from a ruptured pulmonary vascular malformation. Furthermore, compared with diagnostic bronchoscopy, imaging has limitations in diagnosing bronchial mucosal lesions and is unable to provide a histological diagnosis. Consequently, we employed a combined approach, integrating lung-enhanced CT (figure 1) and bedside bronchoscopy (figure 3), to ascertain the presence of a pulmonary vascular malformation and pulmonary embolism.

A large body of evidence-based medical evidence supports the use of ECMO technology in paediatric patients with ARDS and respiratory failure.^4^ The use of ECMO to treat pulmonary haemorrhage is uncommon because ECMO procedures require systemic heparin anticoagulation, which carries a risk of exacerbating bleeding. However, there are still some experts who believe that ECMO could be used for acute lung haemorrhage that is difficult to control with conventional treatment. They concluded that early initiation of ECMO improves oxygenation in pulmonary haemorrhage and buys time for treatment.^5^ The majority of reviews or reports of cases of pulmonary haemorrhage treated with ECMO support the use of ECMO techniques in the treatment of pulmonary haemorrhage and suggest that the risk of bleeding is not necessarily increased during ECMO therapy.^5^,^9^ For example, Hu *et al*,^7^ Kimura *et al*^8^ and Wang *et al*^9^ each reported a case of severe pulmonary haemorrhage treated with maintenance systemic heparin anticoagulation during ECMO surgery without an increased risk of bleeding in children. This suggests that ECMO is not a contraindication to the treatment of respiratory failure due to pulmonary haemorrhage but an effective treatment. There have also been cases where routine heparin anticoagulation has not been used in ECMO patients with organ bleeding.^10^,^13^ ECMO without heparin is prone to thrombus formation, as in this case, where the ECMO developed a thrombus occlusion of the tube after approximately 36 hours of operation.^13^ The coagulation status of the organism is a useful indicator for assessing anticoagulation therapy during ECMO surgery. It has been reported that it is relatively safe and effective to adjust the dose of heparin used during ECMO therapy according to the results of the coagulation profile and TEG in the treatment of pulmonary bleeding.^14^ Therefore, in this case too, we used a low dose of heparinised running ECMO to treat pulmonary haemorrhage in response to the patient’s coagulation status by monitoring the TEG index. This may be the best option to reduce the rate of adverse events without tube occlusion during ECMO therapy while reducing the risk of pulmonary haemorrhage (table 2).

Currently, the common anticoagulation strategy for children or adults with pulmonary haemorrhage treated with ECMO is to give a one-time loading dose of heparin of 50–100 IU/kg after ECMO operation and then adjust the heparin dose of 10–40 U/kg/hour according to the monitoring of activated clotting time, and at the same time maintain a platelet count of more than 100×10^9^/L and fibrinogen of 2.0 g/L by platelet transfusion or cold precipitation.^15^ Overall, the management of pulmonary haemorrhage, especially major haemorrhage, is critical to control haemorrhage and stabilise the airway, and the cause of death is usually due to haemorrhage and airway obstruction caused by thrombus, leading to asphyxia, cardiac arrest and respiratory and circulatory failure. Therefore, when faced with patients with severe pulmonary contusions and uncontrolled pulmonary haemorrhage being treated with ECMO, it is important that we not only prevent thrombosis during the operation of the ECMO machine but also try not to increase the risk of bleeding.

**Learning points**

- Pulmonary haemorrhage is a critical condition that requires urgent multidisciplinary management.
- Extracorporeal membrane oxygenation (ECMO) has emerged as a highly efficacious therapeutic strategy for addressing respiratory failure arising from pulmonary haemorrhage.
- The judicious use of heparin anticoagulation is very important in the clinical management of pulmonary haemorrhage on ECMO.
